# Anti-*N*-methyl-d-aspartate receptor encephalitis and positive human herpesvirus-7 deoxyribonucleic acid in cerebrospinal fluid: a case report

**DOI:** 10.1186/s13256-023-03909-x

**Published:** 2023-06-29

**Authors:** Viktorija Simonavičiutė, Rūta Praninskienė, Jurgita Grikinienė, Rūta Samaitienė-Aleknienė

**Affiliations:** Faculty of Medicine, Vilnius University, Vilnius, Lithuania

**Keywords:** Anti-NMDAR encephalitis, Children, Seizures, HHV-7

## Abstract

**Background:**

Anti-*N*-methyl-d-aspartate receptor encephalitis is a neuroautoimmune syndrome typically presenting with seizures, psychiatric symptoms, and autonomic dysfunction. Human herpesvirus-7 is often found with human herpesvirus-6 and infects leukocytes such as T-cells, monocytes–macrophages, epithelial cells, and central nervous system cells. The pathogenicity of human herpesvirus-7 is unclear. Cases of anti-*N*-methyl-d-aspartate receptor encephalitis with human herpesvirus-7 present in cerebrospinal fluid have been documented, but the clinical significance of this finding remains unclear.

**Case presentation:**

An 11-year-old Caucasian boy was admitted to hospital after a generalized tonic–clonic seizure. Generalized tonic seizures repeated three more times during the day of hospitalization. Blood tests showed minor ongoing inflammation, while brain computed tomography yielded normal results. Brain magnetic resonance imaging showed hyperintense focal alterations in both temporal lobes, hippocampi, and at the base of the right frontal lobe. Positive anti-*N*-methyl-d-aspartate receptor antibodies were found in both serum and cerebrospinal fluid. Positive novel coronavirus 2 (severe acute respiratory syndrome coronavirus 2) immunoglobulin G antibodies were found in serum. Polymerase chain reaction test for severe acute respiratory syndrome coronavirus 2 was negative. Furthermore, positive human herpesvirus-7 deoxyribonucleic acid was found in cerebrospinal fluid. The patient was treated with acyclovir, human immunoglobulin, and methylprednisolone. The seizures did not repeat, and no psychiatric symptoms were present. The patient made a full recovery.

**Conclusions:**

We present a pediatric case of anti-*N*-methyl-d-aspartate receptor encephalitis with atypical clinical presentation. The role of human herpesvirus-7 in neurological disorders remains unclear in immunocompetent patients.

## Background

Anti-*N*-methyl-d-aspartate receptor (anti-NMDAR) encephalitis is a neuroautoimmune syndrome, in which autoantibodies are formed by various different stimuli and cross-react with synaptic protein *N*-methyl-d-aspartate receptor. Anti-NMDAR encephalitis clinically begins with a prodrome period with fever and respiratory or gastrointestinal tract symptoms. This is followed by a psychotic or seizure phase including emotional and behavioral disturbances, ataxia and choreiform movements, and generalized tonic–clonic seizures. Finally, the disease enters the hyperkinetic phase with cardiac symptoms, dyskinesia, extra-pyramidal signs, and stereotyped motor automatisms [1, 2]. Anti-NMDAR encephalitis symptoms may vary in children compared with adults: children present with more neurologic symptoms, dyskinesias or seizures, rather than psychiatric symptoms [3].

Human herpesvirus-7 (HHV-7) is often found with human herpesvirus-6 (HHV-6) and infects leukocytes such as T-cells, monocytes–macrophages, epithelial cells, and central nervous system (CNS) cells. The pathogenicity of HHV-7 is unclear [4]. Usually asymptomatic primary HHV-7 infection might manifest with viral rashes, unspecific febrile syndrome, and exanthem subitum. HHV-7 by itself might induce encephalitis in immunocompromised patients [5, 6] but whether its primary or secondary infection might manifest as viral HHV-7 encephalitis in an immunocompetent person is unclear.

We present a case of an 11-year-old boy with anti-NMDAR encephalitis with atypical clinical presentation and positive HHV-7 deoxyribonucleic acid (DNA) in the cerebrospinal fluid. We also discuss a possible connection between the two pathologies.

## Case presentation

An 11-year-old Caucasian boy was hospitalized after one generalized tonic–clonic seizure lasting about 30 seconds. Two days prior, the patient had a subfebrile temperature of 37.5 °C, stomach pains, and one vomiting episode. The patient had another generalized tonic seizure at the emergency ward and was immediately hospitalized to the intensive care unit (ICU), where the generalized tonic seizures repeated two more times. Intravenous midazolam (5 mg) was used to abort ongoing seizures.

Previous medical history: 5 months ago, the patient’s parents had severe acute respiratory syndrome coronavirus 2 (SARS-CoV-2) infection. The patient’s father had a positive virus polymerase chain reaction (PCR) test and infection symptoms, while the patient’s mother was not tested but did have symptoms as well. The patient himself did not have any infection symptoms and was not tested for SARS-CoV-2 at the time.

The patient was born on time and his early development was normal. The patient was vaccinated according to the national vaccination schedule of Lithuania: by the age of 11 years he was vaccinated with vaccines against tuberculosis, hepatitis B, measles, mumps and rubella, diphtheria, tetanus, pertussis, poliomyelitis, hemophilus influenza type B, pneumococcal disease, and type B meningococcal disease. The patient had additional vaccination against tick-borne encephalitis. The patient had a varicella infection at the age of 3 years. The patient was observed by a cardiologist for a functional systolic murmur, and heart pathology was not found. The patient had been diagnosed with myopia in both eyes. Both patient’s parents were healthy, and the patient’s grandmother has diabetes mellitus.

**Fig. 1:**
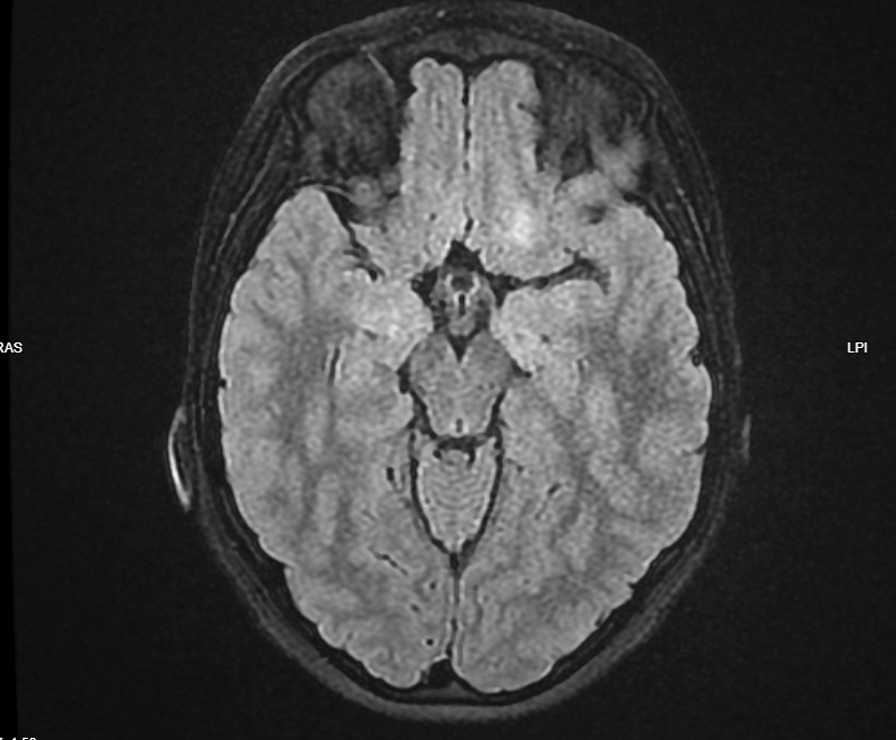
Image of a brain MRI scan, Ax T2 Flair. Image of a brain MRI scan performed during acute phase of a disease showing brain cortex thickening with hyperintense signal in both hippocampi and another 10 mm hyperintense focus at the base of the right frontal lobe

On admission, the patient was somnolent and barely communicated, and his weight was 50 kg. The patient’s vital signs were measured: his body temperature was 36.5 °C, he had stable hemodynamic with a pulse rate of 111 beats/minute, and blood pressure of 130/69 mmHg. The patient was breathing spontaneously with a respiratory rate of 20 breaths per minute, oxygen saturation of 71%, and peroral cyanosis was visible. The patient’s consciousness assessment revealed 9 points on the Glasgow Coma Scale (GCS: eye-opening response 4 points, verbal response 1 point, motor response 4 points). The first neurological examination was performed after the patient was already medicated with midazolam and verbal contact was difficult. During the examination, cranial and bulbar nerves were intact, and no nystagmus was detected. Low, symmetric muscle tone was observed. Deep tendon reflexes were symmetric. No limb paresis was observed. Meningeal symptoms were not present. Blood tests revealed neutrophilic leukocytosis (leukocyte count of 16.87 × 10^9^/l, with a neutrophil count of 9.10 × 10^9^/l), respiratory acidosis with blood pH of 7248, elevated lactate levels (8.1 mmol/l), and elevated blood glucose (6.9 mmol/l). Electrolytes (K, Na, Ca, Cl) and C-reactive protein were within normal ranges. Coagulation assessment and d-dimers were normal. Liver and kidney function blood tests were normal. SARS-CoV-2 PCR was negative; however, immunoglobulin (Ig) G antibodies against the virus were positive (1351.3 AU/ml). A computed tomography (CT) scan of the brain was performed followed by an electroencephalogram (EEG) and brain magnetic resonance imaging (MRI). Brain CT scan yielded normal results, EEG showed focal intermittent theta and delta activity in the left frontal region, possibly caused by inflammation. Brain MRI showed brain cortex thickening with hyperintense signals in both hippocampi and another 10 mm hyperintense focus at the base of the right frontal lobe (Fig. 1). The next day the patient was somnolent (GCS: 13–14), refused to eat, and barely communicated, answering in one word only. No new focal neurological signs were found. Respiratory rate was 16–20 breaths per minute, and oxygen saturation of 92–95%. The seizures did not repeat. In ICU the patient was treated with oxygen 3 l/minute via a mask, intravenous midazolam 5 mg, intravenous dexamethasone 8 mg two times a day, and intravenous 10% mannitol 120 ml two times. After 2 days of treatment in ICU, the patient’s condition stabilized, consciousness and speech normalized, and he was transferred to the children’s neurology ward.

Suspecting encephalitis, appropriate serum and cerebrospinal fluid (CSF) tests were performed: herpes simplex virus 1/2 DNA, herpes 6 virus DNA, and herpes 8 virus DNA were negative; IgM and IgG antibodies against varicella-zoster, Lyme disease, and tick-borne encephalitis were negative (with a few exceptions of a positive IgG of tick-borne encephalitis after vaccination and IgG against varicella-zoster after varicella infection). CSF testing revealed no pleocytosis, no alterations in immunoglobulin G and M or albumin levels, and no intrathecal IgG synthesis. His glucose concentration was elevated (4.3 mmol/l) in CSF and in the blood (6.2 mmol/l).

Testing serum and CSF for autoimmune encephalitis markers showed positive antibodies against NMDA receptors (by semi-quantitative cell-based indirect fluorescent antibody assay). Positive HHV-7 DNA in the CSF was found. After positive autoimmune encephalitis diagnosis, the treatment with intravenous human immunoglobulin 2 g/kg for 5 days and intravenous methylprednisolone 1000 mg/day was started. During the hospitalization, the treatment was continued with intravenous dexamethasone 8 mg two times a day for 5 days overall, oral oxcarbazepine 150 mg three times a day for 7 days, lowering the dose to 75 mg two times a day for 3 days, intravenous acyclovir 500 mg three times a day for 6 days, intravenous human immunoglobulin 2 g/kg administering 20 g a day for 5 days, and intravenous methylprednisolone 1000 mg for 5 days. No medication was continued after the hospitalization.

**Fig. 2:**
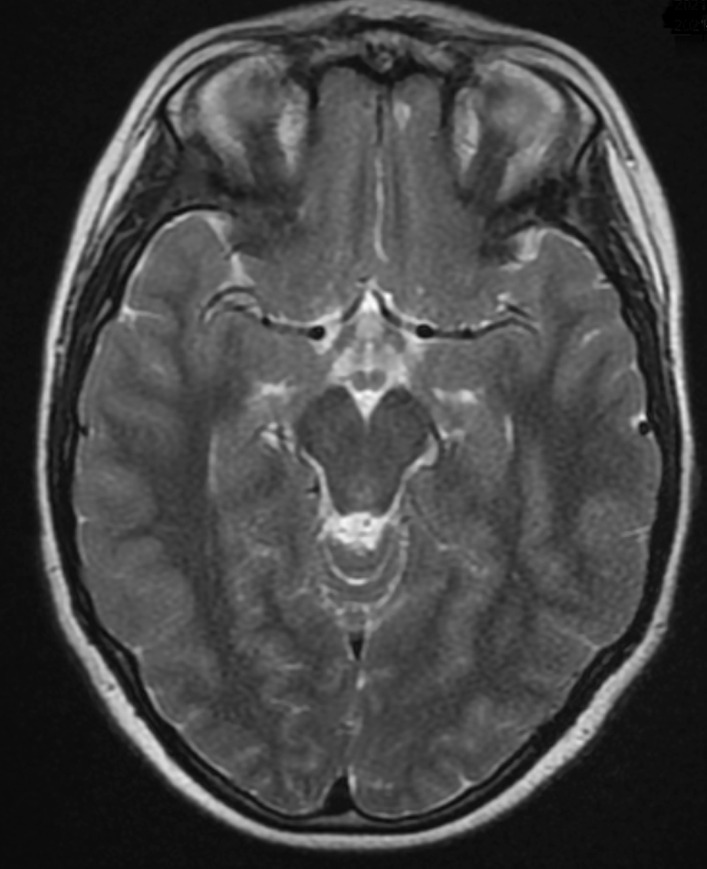
Image of brain MRI scan, Ax T2 FSE. Image of a brain MRI scan performed 3 months after the acute phase. Brain cortex hyperintense signal in both hippocampi and at the base of the right frontal lobe have resolved

The patient was tested for a possible neoplastic source, and ultrasounds of urogenital and digestive systems were without pathology. A control EEG after the treatment was normal and displayed positive disease dynamic. After 3 months, a control brain MRI showed no abnormal signal (Fig. 2). Knowing that anti-NMDAR encephalitis is associated with psychiatric symptoms and cognitive changes [2], the patient was examined by a medical psychologist and no cognitive changes were observed.

Follow-up assessment a year later by a family doctor showed no abnormal physical, mental, or blood examination results. Another brain CT scan was performed 1 year and 8 months later (the patient had an unrelated minor head trauma) and showed no abnormal signal.

Previous medical history: 5 months ago the patient’s parents had SARS-CoV-2 infection, the patient’s father had a positive virus PCR test and infection symptoms, while the patient’s mother was not tested but did have symptoms as well. The patient himself did not have any infection symptoms and was not tested for SARS-CoV-2 at the time.

The patient was born on time and his early development was normal. The patient was vaccinated according to the national vaccination schedule of Lithuania, by age 11 years he was vaccinated with vaccines against tuberculosis, hepatitis B, measles, mumps and rubella, diphtheria, tetanus, pertussis, poliomyelitis, hemophilus influenza type B, pneumococcal disease, and type B meningococcal disease. The patient had additional vaccination against tick-borne encephalitis. The patient had a varicella infection at the age of 3 years. The patient was observed by a cardiologist for a functional systolic murmur, and heart pathology was not found. The patient had been diagnosed with myopia in both eyes. Both patient’s parents were healthy, and the patient’s grandmother has diabetes mellitus.

## Discussion

The case of anti-NMDAR encephalitis described in this article showed an acute disease dynamic. The disease started with subfebrile fever and gastrointestinal symptoms, and after 2 days patient developed recurrent seizures with altered consciousness and speech disturbance for 2 days. There were no common psychiatric or behavioral symptoms present. We faced three main diagnostic problems.

First diagnostic problem: the confirmation of anti-NMDAR encephalitis was difficult, considering the atypical clinical symptoms.

According to multiple studies, anti-NMDAR is more prevalent in female patients and patients younger than 20 years [7–10]. Male patients are more common in age groups bellow 12 years and above 45 years, compared with other age groups, and a neoplastic source of autoimmune encephalitis is less common in those age groups [11]. The main cause of anti-NMDAR encephalitis in women is ovarian teratoma; however, in children and men, it can develop after infections and other stimuli [8]. Anti-NMDAR encephalitis symptoms may vary in children compared with adults. After the nonspecific prodrome of fever and respiratory or gastrointestinal tract symptoms, teenagers and adults develop psychiatric symptoms, which may be difficult to differentiate from a psychiatric disease [8]. Our patient presented with seizures but did not manifest psychiatric symptoms or cognitive changes. The diagnosis was confirmed by the detection of anti-NMDAR antibodies in serum and CSF. A multi-institutional observational study [11] showed that children with anti-NMDAR encephalitis are more likely to experience seizures compared with adult patients [238 adults (65%) presented with behavioral problems, while 55 children below 12 years (50%) presented with seizures or movement disorders].

In 2016, a group of experts provided diagnostic criteria for possible autoimmune encephalitis, which include the subacute onset of working memory, altered mental status, or psychiatric symptoms, and at least one of the following—new focal CNS findings, seizures not explained by a previously known seizure disorder, CSF pleocytosis, or MRI features suggestive of encephalitis, and reasonable exclusion of alternative causes. Diagnostic criteria for anti-NMDAR encephalitis can be made when three criteria are met—rapid onset of at least four of the major groups of clinical symptoms (abnormal behavioral or cognitive dysfunction, speech dysfunction, seizures, movement disorder, decreased level of consciousness, autonomic dysfunction, or central hypoventilation), at least one of the following laboratory study results (abnormal EEG, CSF with pleocytosis or oligoclonal bands), and reasonable exclusion of other disorders [12]. The patient described in this article presented with rapid onset of three out of six major clinical symptom groups: seizures, decreased level of consciousness, and speech dysfunction (verbal reduction) for 2 days. The abnormal laboratory results were identified (abnormal EEG and MRI) but CSF findings were without pleocytosis or intrathecal IgG synthesis.

According to the international consensus treatment recommendation for pediatric NMDA receptor antibody encephalitis, intravenous corticosteroids with additional intravenous immunoglobulin or plasma exchange is recommended as treatment in all children. First-line immunotherapy includes intravenous or oral methylprednisolone (20–30 mg/kg/day for 3–5 days), oral dexamethasone as an alternative to intravenous corticosteroids (20 mg/m^2^/day for 3 days), intravenous immunoglobulin (2 g/kg over 2–5 days), and therapeutic plasma exchange in severe cases. Second-line treatment should be considered after 2 weeks if the initial treatment is not effective. Second-line immunotherapy includes intravenous rituximab, cyclophosphamide, and tocilizumab [13]. Our patient responded well to first-line treatment and recovered within a few days, and no additional treatment was needed after the hospitalization.

Second diagnostic problem: the role of positive HHV-7 DNA in the cerebrospinal fluid of the patient was unclear, and a possible connection between the two pathologies was unclear.

A study of Pohl-Koppe *et al*. investigated whether HHV-7 affects CNS in the absence of exanthem subitum. One study [5] investigated CSF samples of 68 children with CNS diseases compared with a control of 26 children with infectious diseases but without neurological diseases. Six out of 68 children examined had HHV-7 DNA in CSF, compared with none in the control group. The study concluded that HHV-7 infection might be connected to central nervous system disease. Another study [14] examined the CSF of immunocompetent adult patients with neurological disease looking for HHV-7 DNA and a possible connection between neurological symptoms, and the presence of a virus in CSF. Out of a total of 251 patients, HHV-7 DNA was found in the CSF of 14 patients. Furthermore, antiviral treatment reduced neurological symptoms. Those studies suggest that HHV-7 DNA in CSF might not be an irrelevant finding. Described neurological diseases associated with HHV-7 were CNS infections, noninfectious neurological disorders, and among children, facial palsy, vestibular neuritis, and febrile seizure. A study of Mexican pediatric patients with anti-NMDAR encephalitis found that 6 of 31 patients had human herpesviruses detected in CSF, in five of those patients HHV-7 was found. All patients within the study that had positive human herpesviruses had seizures during the disease and none of them had typical symptoms associated with herpetic encephalitis. This article suggests that patients with HHV-7 or HHV-6 detected in CSF might have epileptic seizures more resistant to treatment, but the functional prognosis of these patients was positive [15]. Whether HHV-7 might induce anti-NMDAR encephalitis remains unknown.

The patient described in this case did not present any symptoms that could be associated with HHV-7 infection, and unfortunately, there were no tools available to assess possible ongoing asymptomatic infection by measuring the antibodies against HHV-7 infection either in serum or CSF. The patient had received antiviral treatment in form of intravenous acyclovir as a prevention measure, but it is not quite known whether acyclovir was effective in relieving the symptoms or whether it was the other administered treatment, knowing that foscarnet and ganciclovir are more effective [16] against the virus. According to available literature, the findings of HHV-7 DNA in CSF can lead to more serious neurologic conditions and more difficult recovery; however, in our case, the patient reacted to treatment well, and was fully cured within a few weeks, with no additional post-hospitalization treatment necessary [5, 14, 15].

Third diagnostic problem in our patient: the exclusion of alternative causes was mandatory.

The differential diagnosis of this case clinical presentation includes viral encephalitis, with primary considerations being human herpesvirus simplex and Epstein–Barr viruses [17]. Lyme neuroborreliosis, human herpesvirus simplex, and tick-borne encephalitis were ruled out. Appropriate blood serum and CSF tests were made, and the results showed positive HHV-7 DNA in CSF only. Unfortunately, there was no possibility to assess whether the HHV-7 was responsible for any active or previous infection by checking blood levels of IgM or IgG against HHV-7. The patient was tested for possible neoplastic sources, and ultrasounds of the urogenital and digestive systems were without pathology. Epilepsy was ruled out: the first EEG showed focal intermittent theta and delta activity in the left frontal region and no epileptiform activity. A control EEG after the treatment was normal and the patient displayed a positive disease dynamic with no more seizures during follow-up. Multisystem inflammatory syndrome in children is another rare condition associated with COVID-19 [18]. IgG antibodies against the SARS-CoV-2 virus were found positive in our patient; however, SARS-CoV-2 PCR was negative. In our case, we found no clinical criteria for diagnosing multisystem inflammatory syndrome.

Certain viruses have been associated with the manifestation of autoimmune encephalitis including anti-NMDAR encephalitis, especially within the herpesvirus family—cases of anti-NMDAR encephalitis in adults and teenagers have been documented [19–21]. While there are case reports of the herpes simplex virus triggering anti-NMDAR encephalitis, whether HHV-7 can trigger autoimmune encephalitis remains unknown.

## Conclusions

The case of anti-MNDAR encephalitis presented in this article differs from other cases described in literature by not manifesting with psychiatric symptoms and cognitive changes, but rather with seizures. Most possible causes of the anti-NMDAR encephalitis were excluded. Positive HHV-7 DNA finding in the CSF remains an unknown factor. The seizures did not repeat, and no psychiatric symptoms were present after the treatment. The patient made full recovery.

## Data Availability

All data analyzed during this study are included in this published article and were derived from the archived medical records of Vilnius University Hospital Santaros Klinikos.

## Abbreviations

CNS: Central nervous system
CSF: Cerebrospinal fluid
CT: Computed tomography
DNA: Deoxyribonucleic acid
EEG: Electroencephalography
GCS: Glasgow Coma Scale
HHV-6: Human herpesvirus-6
HHV-7: Human herpesvirus-7
ICU: Intensive care unit
IgG: Immunoglobulin G
IgM: Immunoglobulin M
MRI: Magnetic resonance imaging
NMDAR: *N*-Methyl-d-aspartate receptor
PCR: Polymerase chain reaction
SARS-CoV-2: Novel coronavirus 2
